# Multiple Mechanisms of Tigecycline Resistance in *Enterobacteriaceae* from a Pig Farm, China

**DOI:** 10.1128/Spectrum.00416-21

**Published:** 2021-09-15

**Authors:** Jing Wang, Han Wu, Cai-Yue Mei, Yan Wang, Zhen-Yu Wang, Meng-Jun Lu, Zhi-Ming Pan, Xinan Jiao

**Affiliations:** a Jiangsu Key Laboratory of Zoonosis/Jiangsu Co-Innovation Center for Prevention and Control of Important Animal Infectious Diseases and Zoonoses, Yangzhou University, Yangzhou, China; b Key Laboratory of Prevention and Control of Biological Hazard Factors (Animal Origin) for Agrifood Safety and Quality, Ministry of Agriculture of China, Yangzhou University, Yangzhou, China; USDA-ARS

**Keywords:** tigecycline resistance, *tet*(X), *tet*(A) variant

## Abstract

We isolated eight tigecycline-resistant *Enterobacteriaceae* strains from a pig farm in Shanghai, China, including Escherichia coli (*n* = 1), Proteus cibarius (*n* = 1), and Enterobacter hormaechei (*n* = 6). Two of them (E. coli and P. cibarius) were positive for *tet*(X). E. coli SH19PTE6 contained an IncFIA18/IncFIB(K)/IncX1 hybrid plasmid pYUSHP6-tetX, highly similar to other *tet*(X)-bearing hybrid plasmids from E. coli in China. In P. cibarius SH19PTE4, *tet*(X) was located within a new chromosomal integrative and conjugative element (ICE), ICE*Pci*Chn2, belonging to the SXT/R391 ICE family. All tigecycline-resistant E. hormaechei isolates carried the *tet*(A) variant; cloning and transfer of this *tet*(A) variant into various hosts increased their MICs for tigecycline (4- to 8-fold). Tigecycline resistance observed on a pig farm is mediated by the *tet*(A) variant and *tet*(X) via a plasmid or ICE. The rational use of antibiotics such as doxycycline and surveillance of tigecycline resistance in livestock are warranted.

**IMPORTANCE** As a last-resort antimicrobial agent to treat serious infections, the emergence and spread of tigecycline resistance in *Enterobacteriaceae* and Acinetobacter have raised global concerns. Multiple mechanisms mediate tigecycline resistance in *Enterobacteriaceae*, such as the monooxygenase Tet(X), mutations in Tet proteins, and overexpression of efflux pumps. Although tigecycline is not approved for animals, tigecycline resistance has been observed in Escherichia coli, Proteus cibarius, and Enterobacter hormaechei isolates on a pig farm, mediated by the *tet*(A) variant and *tet*(X) via a plasmid or ICE. The heavy use of tetracyclines such as doxycycline in food-producing animals in China may be the reason for the emergence and transmission of tigecycline resistance.

## OBSERVATION

Tigecycline is used as a last-resort antimicrobial agent for the treatment of serious infections caused by multidrug-resistant bacteria, particularly carbapenem-resistant *Enterobacteriaceae* (1). Tigecycline resistance in *Enterobacteriaceae* has been previously associated with the overexpression of efflux pumps (AcrAB-TolC and OqxAB), a ribosomal S10 protein mutation (*rpsJ*), and mutations in the plasmid-mediated Tet proteins [Tet(A), Tet(K), and Tet(M)] (1). Tet(X) family genes encode flavin-dependent monooxygenases that enzymatically inactivate most tetracyclines, including tigecycline (2). The chromosomally located *tet*(X) and its variant *tet*(X2), which originated from *Bacteroides* species, have been sporadically reported worldwide as conferring tigecycline resistance (3, 4). Recently, the identification of novel plasmid-borne *tet*(X) genes [namely, *tet*(X3) and *tet*(X4)] conferring high level of tigecycline resistance in *Enterobacteriaceae* and Acinetobacter from China in 2019 is of great concern (4). [According to the standards of the nomenclature center, http://faculty.washington.edu/marilynr/, all *tet*(X) variant genes at present can only be designated as *tet*(X).] Mobile *tet*(X) genes [*tet*(X3) and *tet*(X4)] have recently been identified in Acinetobacter spp., numerous *Enterobacteriaceae*, and six other bacterial species (3). To date, *tet*(X) and 14 variants [*tet*(X1) through *tet*(X14)] have been identified and confer differential degrees of tigecycline resistance (5). In 2020, a novel plasmid-mediated efflux pump gene cluster, *tmexCD1-toprJ1*, conferring resistance to multiple drugs, including tigecycline, was identified in Klebsiella pneumoniae strains (6). In this study, we aimed to analyze and elucidate the mechanisms of tigecycline resistance in *Enterobacteriaceae* in a pig farm in China.

On 23 September 2019, 128 samples, including pig feces, pig nasal swabs, feed, pig drinking water, vegetables grown on the pig farm, vegetable field soil, floor swabs from pens, and the shoe soles of workers, were collected from a pig farm in Shanghai, China (Table S1 in the supplemental material). One sample per pig was collected, and no more than five samples were collected from pigs in the same house. The samples were incubated in buffered peptone water (BPW) broth for ∼18 to 24 h and then inoculated onto MacConkey agar with and without 2 mg/liter tigecycline. One isolate per plate was selected and identified using 16S rRNA gene sequencing according to a previously described method (7). A total of 74 *Enterobacteriaceae* isolates, including 45 E. coli, 22 Enterobacter cloacae complex, 3 Citrobacter freundii, 2 Leclercia adecarboxylata, 1 Aeromonas veronii, and 1 Proteus cibarius isolate, were obtained (Table S1 in the supplemental material). All isolates had MICs determined for tigecycline, ampicillin, cefotaxime, meropenem, gentamicin, amikacin, streptomycin, tetracycline, doxycycline, chloramphenicol, florfenicol, nalidixic acid, ciprofloxacin, colistin, fosfomycin, and sulfamethoxazole/trimethoprim using the agar dilution method or the broth microdilution method (limited to colistin and tigecycline). The results were interpreted according to EUCAST (https://www.eucast.org). Among the isolates, eight exhibited an MIC of 4 to 16 mg/liter to tigecycline, including one E. coli, one P. cibarius, and six E. hormaechei isolates obtained from different sources, and also showed resistance to multiple antimicrobial agents (Table 1). The remaining 66 isolates were susceptible to tigecycline with MICs of 0.125 to 0.25 mg/liter. We further screened for the presence of *tet*(X) in tigecycline-resistant isolates by PCR and sequencing (8) and confirmed that two of them (E. coli and P. cibarius) were positive for *tet*(X) [former names, *tet*(X4) and *tet*(X6)] (Table 1). All eight isolates failed to transfer tigecycline resistance to E. coli C600 using conjugation following a previously described protocol (9), but E. coli SH19PTE6 successfully transferred *tet*(X) to DH5α via electroporation.

**TABLE 1:** Characterization of tigecycline-resistant isolates in this study

Strain	Species	Source	MLST^*a*^	Resistance genes^*b*^	Tigecycline MIC (mg/liter)	Other resistance patterns^*c*^	Plasmid replicon(s)	Sequencing platform(s)	BioProject accession no.	GenBank accession no. (element)
SH19PTE4	P. cibarius	Shoe sole		*tet*(X)/*tet*(C)/*tet*(H)/*aph(3′)-Ia*/*aadA2*/*strAB*/*floR*/*sul2*/*dfrA32*/*ere*(A)/*hugA*	16	AMP/STR/TET/DOX/CHL/FFC/CL/NAL/CIP/SXT		PacBio	PRJNA724799	MW423608 (ICEPciChn2)
SH19PTE6	E. coli	Pig feces	ST761	*tet*(X)/*tet*(A)/*tet*(M)/*bla*_TEM-1b_/*qnrS1*/*floR*/*sul3*/*dfrA5*/*mef*(B)/*mdf*(A)/*erm*(B)	16	AMP/TET/DOX/CHL/FFC/SXT	IncFIA18, IncFIB(K), IncX1	PacBio	PRJNA724799	MW423609 (pYUSHP6-tetX)
SH19PE20	E. hormaechei	Feed	ST109	*tet*(A)/*bla*_CTX-M-14_/*bla*_ACT-16_/*bla*_LAP-2_/*aac(6′)-IIc/aadA8/strAB/qnrS1/floR/fosA3/sul1/sul2/dfrA12/dfrA14/ere*(A)	8	AMP/CTX/GEN/STR/TET/DOX/CHL/FFC/NAL/CIP/FOS/SXT	IncFIB, IncHI2	Illumina	PRJNA724799	
SH19PE116	E. hormaechei	Feed	ST109	*tet*(A)/*bla*_CTX-M-14_/*bla*_DHA-1_/*bla*_ACT-16_/*bla*_LAP-2_/*aac(6′)-IIc/aadA8/strAB/qnrB4/qnrS1/floR/fosA3/sul1/sul2/dfrA12/dfrA14/ere*(A)	8	AMP/CTX/GEN/STR/TET/DOX/CHL/FFC/NAL/CIP/FOS/SXT	IncFIB, IncHI2	Illumina	PRJNA724799	
SH19PTE2	E. hormaechei	Pig feces	ST109	*tet*(A)/*bla*_CTX-M-14_/*bla*_DHA-1_/*bla*_ACT-16_/*bla*_LAP-2_/*bla*_TEM-1b_/*rmtB*/*aac(6′)-IIc/aadA8/strAB/qnrB4/qnrS1/floR/fosA3/sul1/sul2/dfrA12/dfrA14/ere*(A)	8	AMP/CTX/GEN/AMI/STR/TET/DOX/CHL/FFC/NAL/CIP/FOS/SXT	IncFIB, IncN, IncHI2, Col440l	PacBio, Illumina	PRJNA724799	
SH19PTE3	E. hormaechei	Pig nasal swab	ST109	*tet*(A)/*bla*_CTX-M-14_/*bla*_DHA-1_/*bla*_ACT-16_/*bla*_LAP-2_/*aac(6′)-IIc/aadA8/strAB/qnrB4/qnrS1/floR/fosA3/sul1/sul2/dfrA12/dfrA14/ere*(A)	8	AMP/CTX/GEN/STR/TET/DOX/CHL/FFC/NAL/CIP/FOS/SXT	IncFIB, IncHI2	Illumina	PRJNA724799	
SH19PTE5	E. hormaechei	Pig feces	ST109	*tet*(A)/*bla*_CTX-M-14_/*bla*_DHA-1_/*bla*_ACT-16_/*bla*_LAP-2_/*aac(6′)-IIc/aadA8/strAB/qnrB4/qnrS1/floR/fosA3/sul1/sul2/dfrA12/dfrA14/ere*(A)	16	AMP/CTX/GEN/STR/TET/DOX/CHL/FFC/NAL/CIP/FOS/SXT	IncFIB, IncHI2	Illumina	PRJNA724799	
SH19PTE7	E. hormaechei	Feed	ST200	*tet*(A)/*bla*_CTX-M-3_/*bla*_ACT-16_/*bla*_TEM-1b_/*aac(3)-IId*/*aph(3′)-Ia/aac(6′)-Ib-cr/aadA16/strAB/qnrS1*/*floR/fosA3/arr-3/sul1/sul2/dfrA27/mph(A)/mcr-9*	4	AMP/CTX/GEN/STR/TET/DOX/CHL/FFC/NAL/CIP/FOS/SXT	IncFIB, IncFII_K_, IncN2, IncQ1	Illumina	PRJNA724799	

a MLST, multilocus sequence type.

b Resistance genes located with the *tet*(X) or *tet*(A) variant on the same plasmid or ICE are underlined.

c AMP, ampicillin; CTX, cefotaxime; GEN, gentamicin; AMI, amikacin; STR, streptomycin; TET, tetracycline; DOX, doxycycline; CHL, chloramphenicol; FFC, florfenicol; CL, colistin; NAL, nalidixic acid; CIP, ciprofloxacin; FOS, fosfomycin; SXT, sulfamethoxazole/trimethoprim.

All six tigecycline-resistant E. hormaechei isolates were sequenced using Illumina HiSeq technology, and the sequence reads were assembled into contigs using SPAdes v.3.13.0. The tigecycline-resistant E. coli and P. cibarius isolates, as well as one representative sequence type 109 (ST109) E. hormaechei strain, SH19PTE2, were sequenced using PacBio single-molecule real-time sequencing. The raw sequences were introduced into the nonhybrid Hierarchical Genome Assembly Process (HGAP) v.4. The plasmid sequence(s) or integrative and conjugative element (ICE) structure(s) were analyzed and annotated using ResFinder v.4.1 (https://cge.cbs.dtu.dk//services/ResFinder/), RAST (https://rast.nmpdr.org), MLST (https://cge.cbs.dtu.dk/services/MLST/), PlasmidFinder (https://cge.cbs.dtu.dk/services/PlasmidFinder/), ISfinder (https://www-is.biotoul.fr/), and BLAST (https://blast.ncbi.nlm.nih.gov/Blast.cgi).

**FIG 1:**
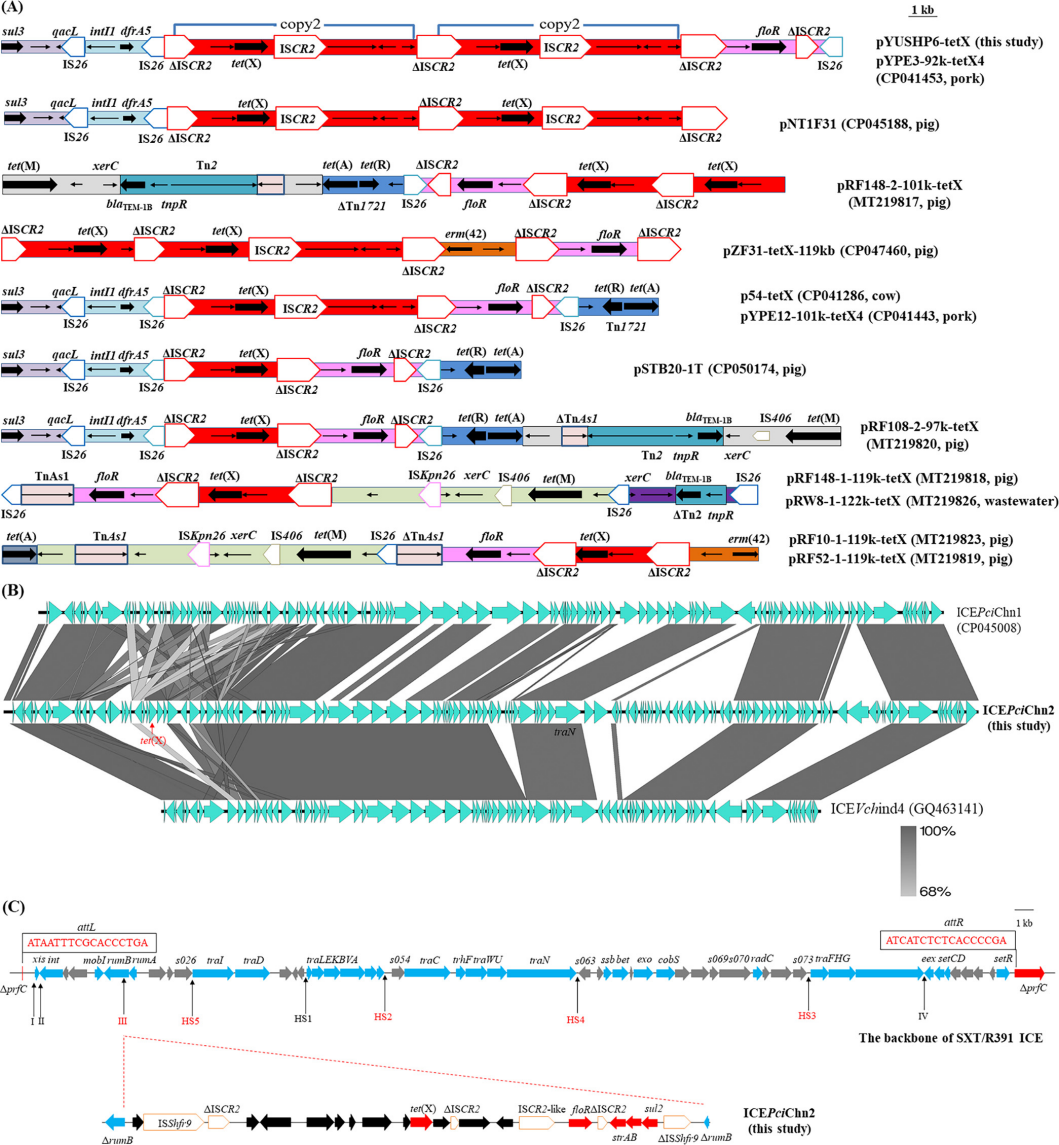
(A) Genetic organization of the *tet*(X) module of pYUSHP6-tetX and comparison with other *tet*(X)-carrying plasmids. The extents and directions of antibiotic resistance (thick arrows) and other genes are indicated. Δ indicates a truncated gene or mobile element. (B) Linear sequence comparison of ICE*Pci*Chn1, ICE*Pci*Chn2, and ICE*Vch*ind4 using Easyfig. The green arrows indicate open reading frames (ORFs). Regions of homology are shaded in gray. (C) Genetic structures of *tet*(X)-bearing ICE in this study. The upper structure shows the standard structure of the SXT/R391 ICE family. The inserted regions (III, HS5, HS2, HS4, and HS3) labeled in red indicate that insertion occurred in ICE*Pci*Chn2 in our study. The *tet*(X) region was inserted into variable region III of the ICE. The red arrows indicate antibiotic resistance genes.

The *tet*(X)-positive E. coli strain SH19PTE6 consisted of a 4,699,374-bp chromosome and three plasmids. Among them, *tet*(X) was located on the largest plasmid, designated pYUSHP6-tetX (GenBank accession no. MW423609). Plasmid pYUSHP6-tetX belonged to the hybrid IncFIA18/IncFIB(K)/IncX1 plasmid with a size of 111,332 bp. It was highly similar in organization to other *tet*(X)-bearing hybrid plasmids from E. coli strains in China, such as plasmids pNT1F31-tetX4 (99.95% identity and 96% coverage) and pZF31-tetX-119kb (99.66% identity and 68% coverage) from pigs, pRW8-1_122k_tetX (97.71% identity and 84% coverage) from wastewater in a swine slaughterhouse, pYPE12-101k-tetX4 (99.96% identity and 99% coverage) from pork, and p54-tetX (99.97% identity and 100% coverage) from a cow (10, 11) (Fig. S1 in the supplemental material). This further highlights the important role of similar IncFIA18/IncFIB(K)/IncX1 hybrid plasmids in the horizontal transfer of *tet*(X). As observed in multiple plasmids, *tet*(X) was associated with the structure ΔIS*CR2*-*orf1*-*abh*-*tet*(X4)-IS*CR2*-*orf2-orf3-orf4*-ΔIS*CR2*, and two copies of this module were present in pYUSHP6-tetX, as found in plasmid pYPE3-92k-tetX4 (Fig. 1A) (10).

The *tet*(X)-positive strain P. cibarius SH19PTE4 harbored one chromosome (4,175,866 bp), and no plasmids were identified. As previously described in P. cibarius strain ZF2 (pig; China) (12), *tet*(X) was located within a chromosomal ICE. SH19PTE4 carried a new ICE, designated ICE*Pci*Chn2 (138,478 bp; GenBank accession no. MW423608), similar to ICE*Vch*ind4 (99.5% identity and 63% coverage) and *tet*(X)-carrying ICE*Pci*Chn1 (98.82% identity and 76% coverage) (Fig. 1B). As it belongs to the SXT/R391 ICE family, ICE*Pci*Chn2 was also integrated into the 5′ end of *prfC* (Fig. 1C). The *tet*(X) gene was embedded in a 30,546-bp region (III) integrated into *rumB* and was probably associated with IS*CR2*, as previously reported (Fig. 1C) (12–14). ICE has become an efficient vector for the transmission of *tet*(X) and tigecycline resistance in Proteus isolates (12–14).

The draft genome sequences of six E. hormaechei isolates were obtained. Isolate SH19PTE2, as a representative ST109 E. hormaechei strain, was further sequenced using PacBio to obtain the whole-genome sequence. We did not identify *tmexCD1-toprJ1* or any genes belonging to the *tet*(X) family in the six E. hormaechei isolates. Compared to the E. hormaechei isolates showing susceptibility to tigecycline in this study, strain FY01 obtained from a patient in France (15), and E. cloacae NCTC9394 (GenBank accession no. FP929040), no amino acid changes within the genes *ramR*, *ramA*, *marA*, *marR*, *acrA*, *acrB*, or *tolC* possibly associated with tigecycline were identified (Table S2 in the supplemental material). However, we found that all tigecycline-resistant E. hormaechei isolates carried the *tet*(A) variant (IncFIB plasmid pYUSHP2-2), identical to that previously described in a tigecycline-resistant K. pneumoniae strain (16). This Tet(A) variant, located within the incomplete transposon Tn*1721*, harbored double frameshift mutations (S201A, F202S, and V203F) and mutations (I5R, V55M, I75V, and T84A), compared to the original Tet(A) in plasmid RP1 (X00006; E. coli). To further confirm its function, the full length of the *tet*(A) variant was amplified using PCR and cloned into the pUC57 vector. The recombinant plasmid pUC57-*tet*(A) variant was transformed into commonly observed *Enterobacteriaceae*, namely, E. hormaechei, E. cloacae, E. coli, Salmonella enterica serovar Typhimurium, and K. pneumoniae. The MICs for tigecycline were increased 4- to 8-fold compared with their host strains (Table S3 in the supplemental material). This further underlines the role of the *tet*(A) variant in tigecycline resistance in various hosts and may explain the tigecycline resistance in the six E. hormaechei isolates in this study.

Although tigecycline has not been approved in animals, tigecycline resistance mediated by the *tet*(A) variant and *tet*(X) via plasmid or ICE was observed on this pig farm. The use of tetracyclines such as doxycycline, one of the most used antimicrobial agents in food-producing animals in China, may be the reason for the emergence and transmission of tigecycline resistance. Appropriate measures should be taken to limit tigecycline resistance in animals.

### Data availability.

The draft genome sequences for the *tet*(X)-bearing plasmid pYUSHP6-tetX and ICE*Pci*Chn2 have been deposited in GenBank under accession no. MW423609 and MW423608. Other sequenced data have been deposited in the GenBank under accession no. PRJNA724799.
