# Supplementary material for: Multiple Mechanisms of Tigecycline Resistance in Enterobacteriaceae from a Pig Farm, China
Source: Microbiol Spectr. 2021 Sep 15;9(2):e00416-21. doi: 10.1128/Spectrum.00416-21 (PMC8557919; doi:10.1128/Spectrum.00416-21)
Supplement: SUPPLEMENTAL FILE 1 — Supplemental material. Download SPECTRUM00416-21_Supp_1_seq4.pdf, PDF file, 1.2 MB [file spectrum00416-21_supp_1_seq4.pdf]

**Table S1. Source and origin of *Enterobacteriaceae* isolates obtained from a pig farm in Shanghai, China**

| Source | No. of samples | No. of isolates (no. of isolates obtained from agar with tigecycline) | | | | | |
|---|---|---|---|---|---|---|---|
| | | *E. coli* | *E. cloacae* complex | *C. freundii* | *L. adecarboxylate* | *P. cibarius* | *A. veronii* |
| Pig feces | 51 | 37(1) | 3(2) | 1 | | | |
| Pig nasal swabs | 10 | 4 | 2(1) | | | | |
| Feed | 24 | 3 | 7(1) | 1 | 2 | | |
| Pig drinking water | 17 | | 3 | | | | 1 |
| vegetables | 5 | | 4 | | | | |
| Vegetable field soil | 5 | | 2 | | | | |
| Floor swabs from pens | 8 | | 1 | | | | |
| Shoe sole from workers | 9 | 1 | | 1 | | 1 | |
| **Total** | **88** | **45** | **22** | **3** | **2** | **1** | **1** |

**Table S2. Comparison between *E. hormaechei* strain SH19PTE2 and other tigecycline susceptible strains. The numbers depict amino acid differences using SH19PTE2 as a reference.**

| strains | species | accession no. | AcrA | AcrB | RamA | RamR | MarR | MarA | TolC |
|---|---|---|---|---|---|---|---|---|---|
| SH19PE4 | *E. hormaechei* | PRJNA724799 | 1 | 0 | 0 | 0 | 0 | 1 | 1 |
| SH19PE5 | *E. hormaechei* | PRJNA724799 | 1 | 0 | 0 | 0 | 0 | 0 | 1 |
| NCTC9394 | *E. cloacae* | FP929040 | 0 | 3 | 0 | 0 | 0 | 0 | 0 |
| FY01 | *E. hormaechei* | PRJNA623478 | 0 | 0 | 0 | 0 | 0 | 0 | 1 |

**Table S3. MICs of tigecycline against parental strains and pUC57-*tet*(A)-variant transformations**

| strains | tigecycline MIC (mg/L) |
|---|---|
| *E. coli* MG1655 | 0.5 |
| MG1655-pUC57 | 0.5 |
| MG1655-pUC57-*tet*(A)-variant | 4 |
| *E. hormaechei* SH19PE5 | 0.5 |
| SH19PE5-pUC57 | 0.5 |
| SH19PE5-pUC57-*tet*(A)-variant | 4 |
| *E. cloacae* CMCC45301 | 0.25 |
| CMCC45301-pUC57 | 0.25 |
| CMCC45301-pUC57-*tet*(A)-variant | 1 |
| *Salmonella* Typhimurium SL1344 | 0.5 |
| SL1344-pUC57 | 0.5 |
| SL1344-pUC57-*tet*(A)-variant | 2 |
| *K. pneumoniae* CMCC46117 | 0.125 |
| CMCC46117-pUC57-*tet*(A)-variant | 1 |

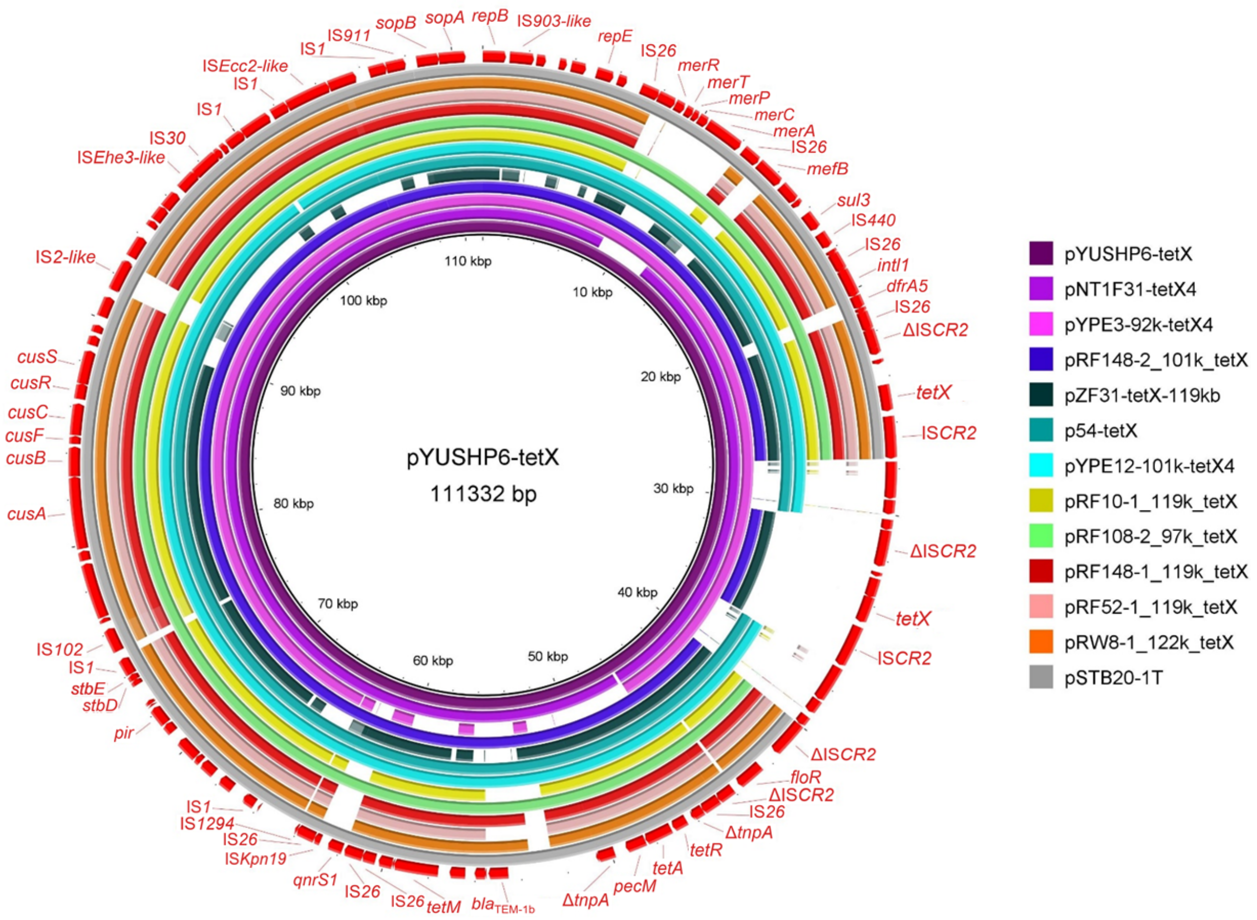


**Figure S1**. Sequence comparison of pYUSHP6-tetX in this study with other IncFIA18/IncFIB(K)/IncX1 plasmids pNT1F31-tetX4 (CP045188, pig), pYPE3-92k-tetX4 (CP041453, pork), pRF148-2_101k_tetX (MT219817, pig), pZF31-tetX-119kb (CP047460, pig), p54-tetX (CP041286, cow), pYPE12-101k-tetX4 (CP041443, pork), pRF10-1_119k_tetX (MT219823, pig), pRF108-2_97k_tetX (MT219820, pig), pRF148-1_119k-tetX (MT219818, pig), pRF52-1_119k_tetX (MT219819, pig), pRW8-1_122k_tetX (MT219826, wastewater), and pSTB20-1T (CP050174, pig) in China using BRIG.
